# Peptidylarginine deiminase 4 deficiency in bone marrow cells prevents plaque progression without decreasing atherogenic inflammation in apolipoprotein E-knockout mice

**DOI:** 10.3389/fcvm.2022.1046273

**Published:** 2022-11-16

**Authors:** Adnana Paunel-Görgülü, Andreas Conforti, Natalia Mierau, Mario Zierden, Xiaolin Xiong, Thorsten Wahlers

**Affiliations:** ^1^Department of Cardiothoracic Surgery, Heart Center, University of Cologne, Cologne, Germany; ^2^Department of Cardiology, Heart Center, University of Cologne, Cologne, Germany; ^3^Center for Molecular Medicine Cologne (CMMC), University of Cologne, Cologne, Germany

**Keywords:** peptidylarginine deiminase 4, atherosclerosis, macrophage polarization, inflammation, bone marrow reconstitution

## Abstract

**Introduction:**

Despite multiple studies in the past, the role of peptidylarginine deiminase 4 (PAD4) in atherosclerosis is currently insufficiently understood. In this regard, PAD4 deletion or inhibition of enzymatic activity was previously reported to ameliorate disease progression and inflammation. Besides, strong influence of neutrophil extracellular traps (NETs) on atherosclerosis burden has been proposed. Here, we studied the role of PAD4 for atherogenesis and plaque progression in a mouse model of atherosclerosis.

**Methods and results:**

Lethally irradiated *ApoE*^–/–^ mice were reconstituted with *ApoE*^–/–^/*Pad4*^–/–^ bone marrow cells and fed a high-fat diet (HFD) for 4 and 10 weeks, respectively. PAD4 deficiency did not prevent the development of atherosclerotic lesions after 4 weeks of HFD. However, after 10 weeks of HFD, mice with bone marrow cells-restricted PAD4 deficiency displayed significantly reduced lesion size, impaired lipid incorporation, decreased necrotic core area and less collagen when compared to *ApoE*^–/–^ bone marrow-transplanted mice as demonstrated by histological staining. Moreover, flow cytometric analysis and quantitative real-time PCR revealed different macrophage subsets in atherosclerotic lesions and higher inflammatory response in these mice, as reflected by increased content of M1-like macrophages and upregulated aortic expression of the pro-inflammatory genes *CCL2* and *iNOS*. Notably, diminished oxLDL uptake by *in vitro*-polarized M1-like macrophages was evidenced when compared to M2-like cells.

**Conclusion:**

These results suggest that pharmacological inhibition of PAD4 may impede lipid accumulation and lesion progression despite no beneficial effects on vascular inflammation.

## Introduction

Atherosclerosis is the dominant cause of cardiovascular disease including myocardial infarction and stroke. It is recognized as being a chronic inflammatory disorder of the vessel wall that is largely driven by the accumulation of pro-inflammatory immune cells, like monocytes, to the endothelial intima, which secrete pathogenic mediators, amplify local inflammation and promote thrombotic complications. Monocytes-derived macrophages represent key effector cells in both early atherogenesis and advanced plaque progression. Within atherosclerotic plaques, macrophages ingest modified lipoproteins and become macrophage-derived foam cells, which promote disease progression (1, 2). Ly6C*^high^* monocytes are considered as precursors of pro-inflammatory M1 macrophages, producing pro-inflammatory cytokines and leading to foam cell formation, whereas Ly6C*^low^* cells were described as resident and patrolling cells, producing anti-inflammatory cytokines and being able to remove damaged cells (3, 4). Recently, it became apparent that Ly6C*^low^* monocytes and reparative macrophages derive from recruited Ly6C*^high^* subsets (5). Both, pro-inflammatory M1 and alternatively activated M2 macrophages have been shown to be present in human (6) and murine (7) atherosclerotic lesions. In humans, M1 macrophages dominate the shoulder regions of the plaque, while the presence of M2 macrophages is associated with vascular adventitia and stable plaques (6, 8). It has further been suggested that M1 macrophages are dominant in vulnerable plaques and enhance atherosclerosis progression in *ApoE^–/–^* (9) and *Ldlr^–/–^* mice (10), whereas polarization toward a M2-like phenotype is associated with atherosclerosis regression (11). However, other studies reported that the majority of aortic macrophages bear markers usually associated with resident and M2-like macrophages (12, 13). In general, stable plaques are characterized by a small lipid core enclosed by a thick fibrous cap, while unstable plaques display a large lipid-reach necrotic core covered by a thin inflamed fibrous cap that prone to rupture (14).

Recently, it has become evident that neutrophils may represent key players in the pathogenesis of atherosclerosis by stimulating the recruitment of inflammatory monocytes and plaque destabilization (15, 16). Activated neutrophils were found to undergo enhanced formation of neutrophil extracellular traps (NETs) at culprit lesion sites (17), consisting of histones, granular and cytoplasmic proteins. NETs were reported to stimulate inflammatory cells, including macrophages (18) and to participate in acute thrombotic complications of intimal lesions (19). Recent data indicate that NETs formation is associated with the appearance of pro-inflammatory M1-like macrophages in atherosclerotic lesions of *Ldlr^–/–^* mice (20).

The enzyme peptidylarginine deiminase 4 (PAD4) is highly expressed in myeloid immune cells such as neutrophils and macrophages and has widely been linked to the regulation of inflammatory processes. By converting arginine residues in histones into citrulline, it plays a fundamental role in NETs formation (21). Indeed, *Pad4^–/–^* mice do not form NETs (22) and myeloid cells-restricted PAD4 deletion was demonstrated to reduce leukocytes recruitment and the expression of pro-inflammatory mediators in atherosclerotic lesions (23). Comparable results could be reproduced in mice treated with the pan-PAD inhibitor chloramidine. However, as chloramidine does not target PAD4 specifically, additional effects on Th2 immune response and dendritic cell maturation, amongst others, can be expected (24, 25).

In the present study, we aimed to investigate the role of PAD4 on the development of atherosclerosis and disease progression using high-fat diet-fed *ApoE^–/–^* mice with bone marrow-restricted PAD4 deficiency. We demonstrate for the first time, that bone marrow-restricted PAD4 deletion impedes lipid accumulation and plaque progression in atherosclerosis despite enhanced accumulation of pro-inflammatory M1-like macrophages and increased signs of inflammation.

## Materials and methods

### Animals

*ApoE*^–/–^ (B6.129P2-Apoetm1Unc/J) mice were purchased from the Jackson Laboratory. The generation of *Pad4^–/–^* mice was previously reported by our group (26). *ApoE^–/–^*/*Pad4^–/–^* mice were generated by crossing *ApoE^–/–^* with *Pad4^–/–^* mice without the Cre recombinase gene. All mice were on a C57/Bl6J genetic background.

Adult 8–12 weeks old male mice (22.86 ± 1.94 g) were used in this study. Mice were maintained in the local animal facility at a 12-h light/dark cycle with food and water *ad libidum*.

### Irradiation, bone marrow transplantation and atherosclerosis model

8–12 weeks old *ApoE^–/–^* mice were subjected to 9-Gy whole body γ-irradiation to eliminate bone marrow cells. Bone marrow cells were extracted from male *ApoE^–/–^/Pad4^–/–^* donor mice by flushing the femurs and tibias. Irradiated mice were injected intravenously with 5 × 10^6^ bone marrow cells in 100 μl PBS. Mice reconstituted with bone marrow cells from *ApoE^–/–^/Pad4^+/+^* (= *ApoE^–/–^*) mice served as control.

Mice were kept on 0.01 mg/ml polymyxin B sulfate and 0.1 mg/ml neomycin, administered through drinking water, for 5 weeks. During the first 4 weeks after transplantation, mice were fed a standard chow diet to allow for bone marrow reconstitution. Afterward, mice were switched to a high-fat diet containing 0.2% cholesterol, 21% butter fat (TD.88137, Ssniff Spezialdiaeten, Soest, Germany) for 4 and 10 weeks, respectively. At the end of experiment, mice were euthanized by terminal anesthesia and blood samples were obtained from the right ventricle. In some groups, animals were fasted for 6 h. The aortic trees including the hearts were immediately dissected and prepared for further analyses. Successful bone marrow reconstitution was verified and confirmed by PCR using gene-specific primers and genomic DNA isolated from blood samples collected at the end of the experiment.

### Aortic digestion and flow cytometry

Aortas were dissected, minced using scissors and enzymatically digested with 200 U/mL *Liberase* (Roche) and 40 U/mL *DNase I* (Sigma Aldrich) in HBSS plus 5% FCS for 1 h at 37°C. Cells were filtered through a 40 μm nylon strainer, washed with HBSS plus 5% FCS, collected by centrifugation at 400 g for 5 min at 4°C and then suspended in FACS buffer (PBS plus 0.2% FCS plus 1 mM EDTA). Murine Fc receptors were blocked using anti-CD16/32 antibodies (BioLegend) for 10 min on ice. Violet 510 Viability Dye (Cell Signaling Technology) was used to discriminate between live and dead cells. The cells were stained with the following antibodies for 30 min at 4°C: PerCP-Cy5.5-conjugated anti-CD45, APC-Cy7-conjugated anti-CD11b, FITC-conjugated anti-Ly6G, PE-Cy7-conjugated anti-F4/80, and APC-conjugated anti-Ly6C (all purchased from BioLegend). Flow cytometric analysis was performed using FACSCanto II flow cytometer (BD Biosciences) and DIVA Software (BD Biosciences).

### Staining of atherosclerotic roots and immunofluorescence

Serial sections (7 μm) of aortic root were collected and stored at -80°C under further processing. Frozen sections were air-dried, fixed with 4% paraformaldehyde for 10 min, stained with Oil Red-O (ORO) for 15 min and counterstained with mayers hematoxylin (two slides per animal). The lesion areas were quantified using Image J software and expressed as mm^2^.

Masson trichrome staining for collagen analysis was performed using Trichome Stains (Masson) (Sigma Aldrich) according to the manufacturer’s protocol. Collagen quantification was determined by the method described by Chen et al. (27). Histological evaluation of necrotic core size was performed on hematoxylin and eosin (H&E-stained sections, two slides per animal).

For immunofluorescence, frozen sections (12–16 sections per animal) were fixed with 4% paraformaldehyde, permeabilized, blocked with 5% serum and stained with the following primary antibodies: rabbit anti-α-SMA (ab5694, Abcam) and anti-CD68 (ab53444, Abcam). Following labeling with the fluorochrome-coupled secondary antibodies Alexa Fluor 488-conjugated goat anti-rabbit IgG (Cell Signaling Technology) and Alexa Fluor 568-conjugated donkey anti-rat IgG (Thermo Fisher), sections were counterstained with DAPI and mounted with fluorescent mounting medium (Dako). All images were captured using an inverted Eclipse Ti-U 100 microscope and NIS Element software package (Nikon). Image J software was used to define the percentage of positive cells.

For NETs staining, frozen sections of aortic root were fixed with 4% paraformaldehyde, followed by permeabilization and blocking with 5% donkey serum. Subsequently, the samples were incubated with goat anti-MPO (R&D Systems) and rabbit anti-citrullinated histone H3 (citH3, Abcam) antibodies, followed by incubation with Alexa Fluor 488-conjuagted anti-goat IgG and Alexa Fluor 555-conjugated anti-rabbit IgG (both from Abcam) for 2 h at room temperature. Tissue sections were counterstained with DAPI and mounted in Dako Fluorescent mounting medium (Dako). NETs were defined as colocalization of MPO and citH3 and visualized using an inverted microscope (Eclipse Ti-U 100, Nikon) and the NIS elements software (BR3.10, Nikon). The percentage of NETs-positive area and percentage of citH3-positive area were calculated using Image J software.

### *En face* staining of aorta

For quantification of atherosclerotic lesions, aorta from root and abdominal area was fixed with 4% paraformaldehyde followed by careful removal of connective tissues. Then, the aorta was opened longitudinally, pinned *en face* and stained with Oil Red-O (ORO) for 3–4 h. Images were taken with a digital camera and both, total surface area and ORO-positive lesion area were determined using Image J software. The extent of atherosclerotic lesion development was defined as the percentage of total ORO-positive lesion area over the total surface area.

### Real-time polymerase chain reaction

Total RNA from dissected aortas and cultured cells was extracted using Trizol (Sigma Aldrich) or the RNeasy Mini Kit (Qiagen), respectively, according to manufacturers’ instructions. RNA was reverse transcribed using High Capacity cDNA Reverse Transcription Kit (Applied Biosystems). Real-time PCR was performed using Power SYBR Green PCR Master Mix (Applied Biosystems) and specific primers for *IL-6*, *IL-12*, *IL-1*β, *MMP-2, MMP-9* (28), *iNOS*, *Ym-1*, *SPHK1*, *LIGHT*, *FIZZ/RELM*-α, *MerTK* (29), *TNF*-α (30), *TGF*-β (31) and *CCL2* (32). All samples were run in triplicates. Expression of target genes was normalized to the GAPDH housekeeping gene and expressed as relative expression (2^Δ^ *^CT^* formula).

### Isolation, culture and polarization of bone marrow-derived macrophages

Bone marrow-derived macrophages from 9 to 12 weeks old *ApoE^–/–^* and *ApoE^–/–^/Pad4^–/–^* mice were prepared by flushing femurs and tibias followed by cell culture in RPMI medium supplemented with 20% FCS and 20 ng/mL murine M-CSF (Peprotech) for 7 days (26). To induce a M1- or M2a-like phenotype, macrophages (M0) were further incubated in the presence of 20 ng/mL IFN-γ (Peprotech) and 100 ng/mL LPS (Sigma Aldrich) or in medium supplemented with 20 ng/mL IL-4 (Peprotech), respectively. Macrophages’ phenotype was characterized by Real-time PCR and flow cytometry (FACS Canto II, BD). For flow cytometry, M0, M1-like and M2a-like macrophages were stained for CD11b, CD86 (M1 marker) and CD206 (M2 marker). The following antibodies were used: APC-Cy7-conjugated anti-CD11b, APC-conjugated anti-CD86 and Brilliant Violet 421-conjugated anti-CD206 (BioLegend).

### oxLDL uptake assay

M1- or M2a-polarized bone marrow-derived macrophages were serum starved for 24 h and further incubated with 20 μg/mL Dil-labeled oxLDL (Themo Fisher) for 6 h. The cells were washed twice with PBS and analyzed by flow cytometry (FACS Canto II, BD).

### Quantification of cholesterol

Plasma cholesterol was measured by standard colorimetric assay kit from Sigma Aldrich (No. MAK043).

### Multiplex assay and cfDNA quantification

Plasma concentrations of IL-6, IL-12, TNF-α and CCL2 were determined by using a customized ProcartaPlex multiplex immunoassay for mouse (ProcartaPlex 4-plex, Thermo Fisher) according to the protocol provided by the manufacturer. Data were collected and analyzed using a Magpix instrument equipped with xPONENT software (Luminex Corporation, Austin, Texas, United States).

cfDNA levels in plasma samples were quantified by Pico green staining as previously described (33).

### Statistical analysis

Experimental data were analyzed with GraphPad Prism software (GraphPad Software, San Diego, CA, USA) and are reported as mean ± standard deviation (SD). Normal distribution of variables was tested using the Kolmogorov-Smirnov test. Comparisons of two groups were analyzed using unpaired *t*-test or the non-parametric Mann-Whitney *U*-test. Differences between groups of two independent variables were determined using two-way ANOVA and Tukey’s *post hoc* test. A *p*-value of less than 0.05 was considered as statistically significant.

## Results

### Peptidylarginine deiminase 4 deficiency prevents neutrophil extracellular traps formation and reduces atherosclerotic lesions and lipid accumulation

**FIGURE 1:**
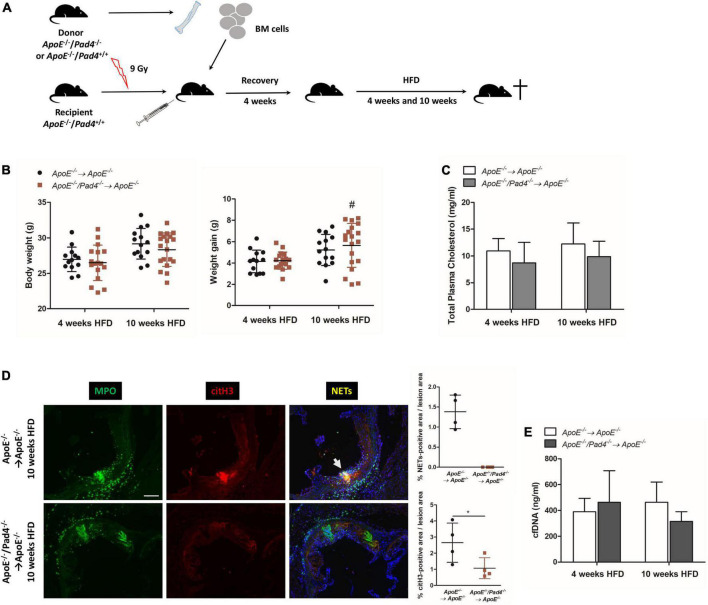
Impact of bone marrow-restricted PAD4 deficiency on body weight and NETs formation after high-fat diet. **(A)** Schematic of the experimental design. **(B)** Body weight and weight gain of mice fed a high-fat diet (HFD) for 4 or 10 weeks, respectively. *n* = 13–19/group. **(C)** Plasma cholesterol levels after HFD. *n* = 4–8/group. **(D)** Representative immunofluorescence images of aortic root sections from mice on HFD for 10 weeks stained for MPO (*green*) and citrullinated histone H3 (*red*) representing NETs (*yellow*). Representative images from four mice per group. Scale bar: 100 μm. Percentage of NETs-positive and citH3-positive area were calculated. *n* = 4/group. **(E)** cfDNA was quantified in plasma of mice after 4 and 10 weeks of HFD, respectively. *n* = 8/group. ^#^*p* < 0.05 vs. 4 weeks HFD; *p < 0.05.

To study the role of PAD4 in bone marrow-derived cells in atherosclerosis, *ApoE^–/–^* mice were subjected to lethal irradiation and reconstituted with bone marrow from *ApoE^–/–^/Pad4^–/–^* or *ApoE^–/–^* mice as illustrated in Figure 1A. Challenging of *ApoE^–/–^/Pad4^–/–^* bone marrow-reconstituted mice with HFD for 10 weeks resulted in a significant increase of body weight gain when compared to mice fed a HFD for 4 weeks and no differences in body weight could be observed between the experimental groups (Figure 1B). Further analysis revealed widely comparable plasma cholesterol levels, indicating that PAD4 does not affect plasma cholesterol (Figure 1C).

Impaired NETs formation in PAD4-deficient mice was already well documented by several groups (19, 22, 23). However, to confirm that bone marrow-restricted PAD4 deficiency impedes NETs formation, we performed immunofluorescent staining to detect colocalization of citrullinated histone 3 (citH3) and myeloperoxidase (MPO) (= NETs) in aortic root lesions. Consistent with previous reports (19, 23), NETs formation was clearly evident in control mice, but not in aortic root lesions of *ApoE^–/–^/Pad4^–/–^* bone marrow-transplanted mice after 10 weeks of HFD feeding (Figure 1D). Moreover, reduced histone H3 citrullination, confirming impaired PAD activity, could be observed.

Plasma cfDNA levels have been proposed to represent biomarkers of NETs release and tissue damage in cardiovascular disease (34). Quantification of plasma cfDNA levels did not reveal any differences between the experimental groups, suggesting that circulating cfDNA does not reflect NETs formation (Figure 1E). Nevertheless, at 10 weeks, cfDNA levels were slightly reduced in mice with bone marrow-restricted PAD4 deficiency.

**FIGURE 2:**
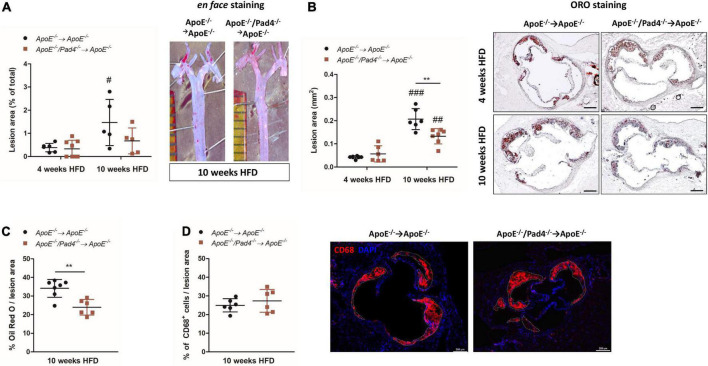
PAD4 deficiency diminishes lesion size and lipid accumulation. **(A)** Quantification of lesion area of *en face*-stained aortas after 4 and 10 weeks of high-fat diet (HFD). Representative ORO-stained aortas after 10 weeks are depicted. *n* = 5–8/group. **(B)** Quantification of lesion area in ORO-stained aortic root sections. Representative images for each time point and each experimental group are depicted. Scale bar: 200 μm. *n* = 6–7/group. **(C)** Quantification of ORO-positive areas in aortic roots. *n* = 6–7/group. **(D)** Quantification of CD68-positive cells (inflammatory macrophages/monocytes) in aortic roots of mice fed a HFD for 10 weeks. Representative images are depicted. Scale bar: 200 μm. *n* = 6/group. ^#^*p* < 0.05, ^##^*p* < 0.01, ^###^*p* < 0.001 vs. 4 weeks HFD; **p < 0.01.

Analyses of *en face*-stained aortas showed significant increase of lesion area in *ApoE^–/–^* control mice after 10 weeks of HFD when compared with the 4 weeks cohort (Figure 2A). However, plaque burden did not differ between mice with bone marrow-restricted PAD4 deficiency and fed a HFD for 4 and 10 weeks respectively, suggesting that PAD4 deficiency impedes plaque progression in atherosclerosis.

Likewise, at 10 weeks, lesion size in aortic root sections of *ApoE^–/–^/Pad4^–/–^* bone marrow-reconstituted mice was significantly reduced compared to the *ApoE^–/–^* control group, although lesions detected after 10 weeks of HFD were generally larger than after 4 weeks (Figure 2B). Notably, reduced lesion size in PAD4-deficient mice was associated with impaired lipid accumulation after 10 weeks of HFD, as demonstrated by reduced ORO-positive lesion area (Figure 2C). This reduction was not related to a decreased number of recruited macrophages, as no differences in CD68-positive cells were detected between both groups (Figure 2D).

**FIGURE 3:**
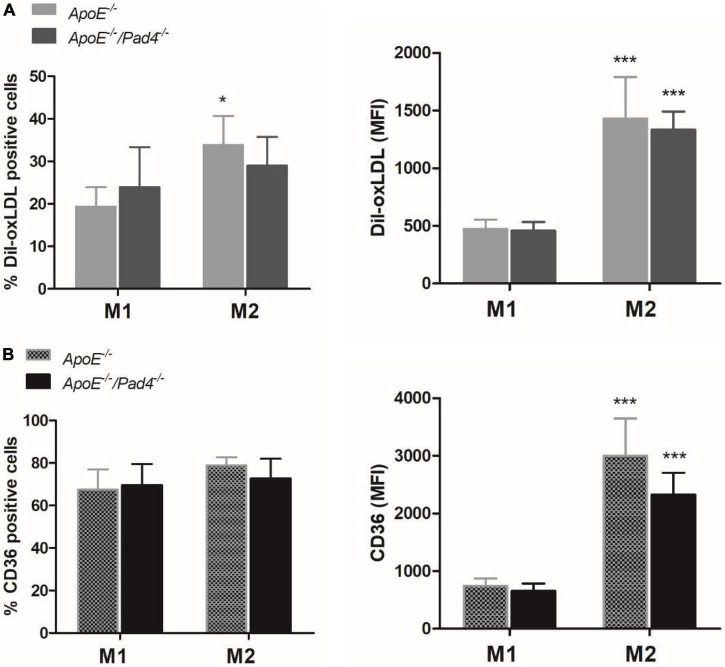
oxLDL incorporation by polarized *ApoE^–/–^* and *ApoE^–/–^/Pad4^–/–^* macrophages *in vitro.* Bone marrow-derived macrophages were polarized *in vitro* with 20 ng/mL IFN-γ and 100 ng/mL LPS or IL-4 to induce a M1- or M2a-like phenotype, respectively. **(A)** Cells were further incubated with Dil-labeled oxLDL for 6 h and internalized oxLDL was quantified by flow cytometry. **(B)** The expression of the oxLDL receptor CD36 was analyzed in parallel. The percentage of positive cells as well median fluorescence value (MFI) are depicted. *n* = 5 *p < 0.05, ***p < 0.001 vs. M1-like macrophages.

The phenotype of macrophages was previously reported to influence the ability of lipid accumulation in atheorosclerosis (35). We therefore next questioned if PAD4 deficiency affects macrophage lipid burden in dependence on their polarization state. For this, bone marrow-derived *in vitro* polarized M1- and M2a-like *ApoE^–/–^* and *ApoE^–/–^/Pad4^–/–^* macrophages were incubated with Dil-labeled oxLDL for 6 h and lipid accumulation was quantified by flow cytometry. As shown in Figure 3A, M2a-like macrophages were found to internalize higher amounts of oxLDL compared to M1-like cells independent on macrophages’ genotype. However, the percentage of *ApoE^–/–^/Pad4^–/–^* M2a-positive cells was somehow lower than of *ApoE^–/–^* M2a macrophages and did not significantly increase. Enhanced oxLDL uptake by M2a-like cells was found to correlate with increased expression of the oxLDL receptor CD36 (Figure 3B) and no differences between both genotypes could be observed. These results suggest that the ability to accumulate modified lipids is markedly higher in M2 like-macrophages compared to M1-like cells, while no substantial influence by PAD4 deficiency became evident.

### Bone marrow cells-restricted peptidylarginine deiminase 4 deficiency is associated with reduced necrosis and collagen production

**FIGURE 4:**
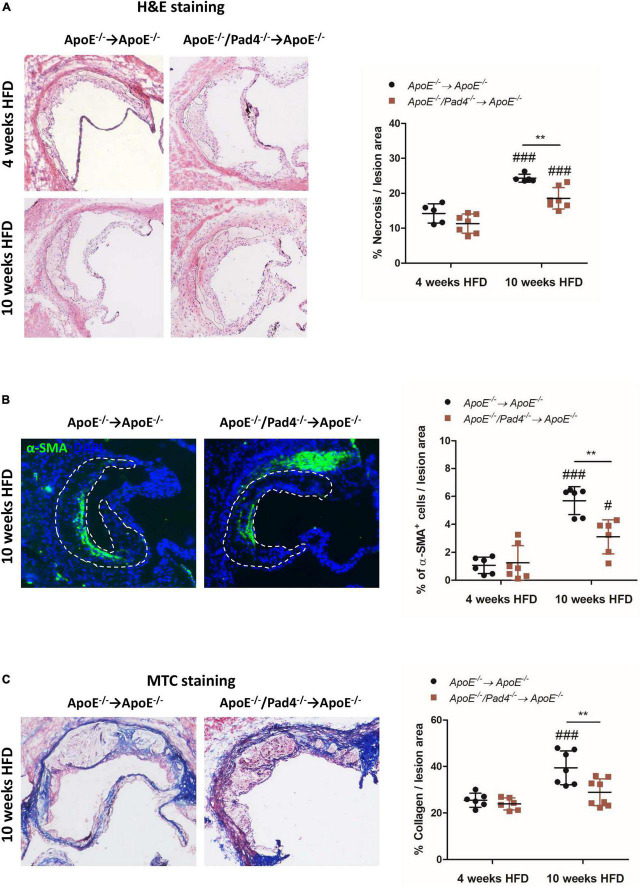
PAD4 deficiency in bone marrow cells results in increased stability of atherosclerotic lesions. **(A)** Quantification of necrotic core area in H&E-stained aortic root sections of mice fed a high-fat diet (HFD) for 4 or 10 weeks, respectively. *n* = 5–7/group. **(B)** Quantification of α-SMA-positive area (smooth muscle cells) in atherosclerotic lesions of aortic root. Representative images are shown. *n* = 6–7/group. **(C)** Masson’s Trichrome staining of aortic root sections for collagen quantification. Representative images for each experimental group are depicted. *n* = 6–8/group. ^#^*p* < 0.05, ^###^*p* < 0.001 vs. 4 weeks HFD, **p < 0.01.

Plaque vulnerability and the risk of rupture highly depend on necrotic core size, collagen content as well as inflammation. Necrotic core size increased significantly in both experimental groups after 10 weeks of HFD compared to the size determined after 4 weeks (Figure 4A). However, after 10 weeks, necrotic core areas in *ApoE^–/–^/Pad4^–/–^*-reconstituted mice were found to be significantly decreased compared to *ApoE^–/–^* control mice, indicating that PAD4 deficiency is associated with reduced cell death.

Additionally, at the same time point, *ApoE^–/–^/Pad4^–/–^*-reconstituted mice showed reduced abundance of α-SMA^+^ smooth muscle cells (SMCs) (Figure 4B), which are considered to have a beneficial role by contributing to the stabilization of atherosclerotic lesions (36). Accordingly, reduced collagen content in lesions of *ApoE^–/–^/Pad4^–/–^* bone marrow-transplanted mice was observed, as determined by Masson’s Trichrome staining (Figure 4C). We further found the number of α-SMA^+^ cells as well as collagen-positive area to strongly increase in *ApoE^–/–^*-reconstituted control mice after 10 weeks of HFD when compared to the earlier time point (4 weeks). However, this increase was abrogated in *ApoE^–/–^/Pad4^–/–^*-transplanted mice. Altogether, these data indicate that PAD4 deficiency diminishes the abundance of collagen-producing SMCs.

### Bone marrow cells-restricted peptidylarginine deiminase 4 deficiency influences the phenotype of intralesional macrophages and vascular inflammation

**FIGURE 5:**
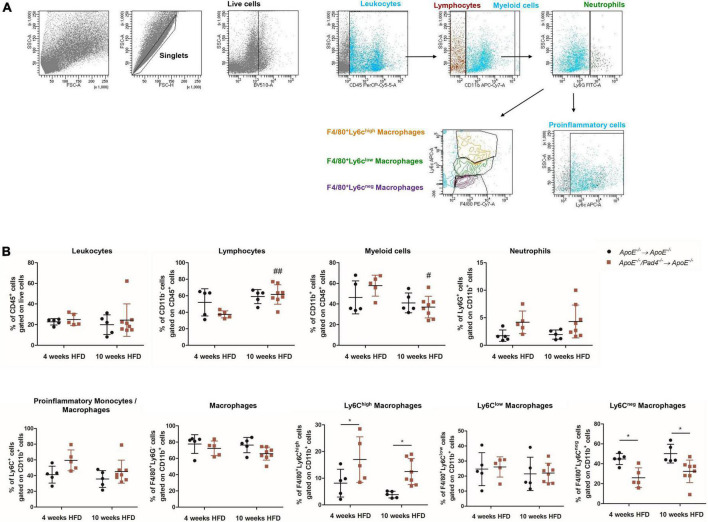
PAD4 modulates macrophages’ phenotype *in vivo*. Aortas from mice were enzymatically digested and cells were stained with different fluorochrome-conjugated antibodies followed by flow cytometric analysis. **(A)** Representative plots illustrating the gating strategy to identify immune cell populations. **(B)** Quantification of leukocytes (CD45^+^), lymphocytes (CD45^+^CD11b^–^), myeloid cells (CD45^+^CD11b^+^), neutrophils (CD45^+^CD11b^+^Ly6G^+^), inflammatory monocytes/macrophages (CD45^+^CD11b^+^Ly6G^–^Ly6C^+^), macrophages (CD45^+^CD11b^+^Ly6G^–^F4/80^+^) as well as M1-like (F4/80^+^Ly6C^high^) and M2-like (F4/80^+^Ly6C^low^ and F4/80^+^Ly6C^neg^) macrophages in digested aortas from mice after 4 and 10 weeks of high-fat diet (HFD). *n* = 5–8/group. ^#^*p* < 0.05, ^##^*p* < 0.01 vs. 4 weeks HFD; *p < 0.05.

Given that atherosclerotic burden is mainly driven by inflammatory processes (37), we next determined the impact of PAD4 deficiency on the recruitment of immune cells to the lesions by performing flow cytometric analysis. Gating strategy is depicted in Figure 5A. The percentage of recruited CD45^+^ leukocytes in bone marrow-reconstituted mice was comparable (Figure 5B). After 10 weeks of HFD, mice transplanted with *ApoE^–/–^/Pad4^–/–^* bone marrow showed significantly increased number of lymphocytes and reduced content of myeloid cells vs. mice fed a HFD for 4 weeks. Among myeloid cells, no alterations in neutrophil and macrophage contents could be observed. However, PAD4 was found to influence the phenotype of lesional macrophages. Newly recruited inflammatory monocytes (Ly6C^high^) differentiate into Ly6C^high^ macrophages which in turn may convert into anti-inflammatory Ly6C^low^ macrophages through phenotypic switching (5). Based on the expression of F4/80 and Ly6C, three different macrophage subsets were identified, characterized by high, low or absent Ly6C expression. At both time points (4 and 10 weeks HFD), *ApoE^–/–^/Pad4^–/–^*-reconstituted mice displayed significantly higher content of F4/80^+^/Ly6C^high^ macrophages (M1-like) and strongly reduced number of F4/80^+^/Ly6C^neg^ cells, reflecting resident macrophages (M2-like) when compared to *ApoE^–/–^* control mice. The number of F4/80^+^/Ly6C^low^ macrophages did not differ between groups. As the content of Ly6C^+^ inflammatory monocytes/macrophages visibly increased in lesions with PAD4 deficiency, we assume that more recruited Ly6C^+^ monocytes become pro-inflammatory, M1-like macrophages. Moreover, conversion into Ly6C^low/neg^ macrophages seems to be impaired.

As these data demonstrate altered distribution of macrophage subsets in PAD4-deficient lesions, we further aimed to assess the consequences of PAD4 knockout on the expression of macrophage phenotype marker genes using *in vitro*-polarized macrophages. Of note, M1-like *ApoE^–/–^/Pad4^–/–^* macrophages showed significantly higher expression of *IL-6* and *iNOS* as well as a moderate, not significant, upregulation of *TNF*-α expression compared to *ApoE^–/–^* M1-like macrophages. No differences regarding the expression of the M2-specific genes *Ym-1*, *Fizz-1/RELM*-α*1*, *LIGHT*, *SPHK1* and *MerTK* were found between M2a-polarized macrophages (Supplementary Figure 1A). Moreover, increased expression of inflammatory genes in M1-polarized *ApoE^–/–^/Pad4^–/–^* macrophages was accompanied by elevated expression of the costimulatory molecule CD86 (Supplementary Figure 1B), suggesting that PAD4 deletion increases the activity of M1 macrophages.

**FIGURE 6:**
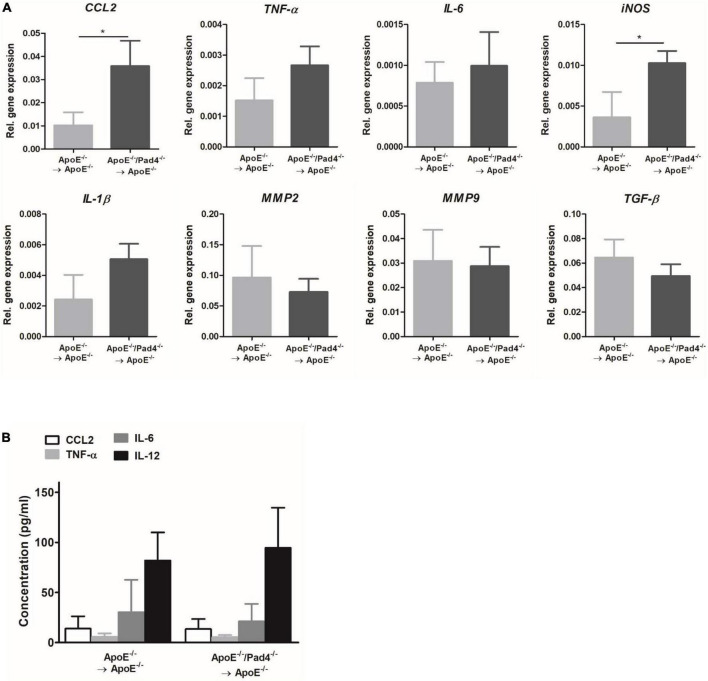
Impact of bone marrow cells-restricted PAD4 deficiency on vascular and systemic inflammation. **(A)** Expression of the pro-inflammatory genes *CCL2*, *TNF*-α, *IL-6*, *iNOS*, *IL-1*β and the fibrosis-related genes *MMP2*, *MMP9* and *TGF*-β in the aortas of mice fed a high-fat diet (HFD) for 10 weeks. *n* = 4/group. **(B)** Quantification of CCL2, IL-6, TNF-α and IL-12 in plasma samples after 10 weeks of HFD by Multiplex assay. *n* = 8/group *p < 0.05.

Having shown that atherosclerotic lesions of mice with PAD4 deficiency are enriched with M1-like macrophages, we next analyzed the expression of inflammatory mediators in mice aortas after 10 weeks of HFD by Real-time PCR (Figure 6). PAD4 deficiency resulted in significantly elevated mRNA expression of the monocytes-attracting chemokine *CCL2* and the pro-inflammatory gene *iNOS* (Figure 6A). Besides, a slight not significant increase of *TNF*-α, *IL-6*, and *IL-1*β could be observed. Conversely, the expression of the collagen-degrading matrix metalloproteinases *MMP2* and *MMP9* and the pro-fibrotic gene *TGF*-β did not differ. Moreover, plasma levels of CCL2, TNF-α, IL-6, and IL-12 were quantified by multiplex assay and were found to be comparable between both groups (Figure 6B). Thus, bone marrow-restricted PAD4 deficiency does not influence systemic inflammation. Taken together, these findings indicate that PAD4 deficiency strongly affects macrophages’ phenotype resulting in a pro-inflammatory state.

## Discussion

NETs and PAD4 have been proposed to be involved in the progression of atherosclerosis. Indeed, PAD4 inhibition by chloramidine was shown to reduce atherosclerosis burden in a mouse model of atherosclerosis (38). Although the protective effects are supposed to be based on NETs deficiency, additional PAD4-dependent effects cannot be doubtlessly excluded. In this study, we studied the role of PAD4 for the development of atherosclerosis and disease progression using irradiated *ApoE^–/–^* mice reconstituted with *ApoE^–/–^/Pad4^–/–^* bone marrow cells and challenged with a high-fat diet.

We found that bone marrow cells-restricted PAD4 deletion strongly impedes the progression of atherosclerosis, although it did not prevent the development of atherosclerotic plaques. These findings are in line with a previous report demonstrating reduced atherosclerosis burden in *ApoE^–/–^* mice with specific PAD4 deletion in the myeloid lineage (23) and further support the prominent role of PAD4 in lesion progression. Comparable results could not be reproduced in *Ldlr^–/–^* mice with bone marrow cells-restricted PAD4 deficiency (19).

In contrast to the data reported by Liu et al. (23), PAD4 deficiency did not impede leukocyte recruitment to atherosclerotic lesions. However, after 10 weeks of HFD significant increase of lymphocytes and reduced content of myeloid cells was observe when compared to the earlier stage of disease. The number of recruited neutrophils was not altered by PAD4 deficiency, but tended to be higher than in *ApoE^–/–^* bone marrow-reconstituted control mice. Previous studies reported that components secreted by neutrophils, like NETs, enhance the recruitment of immune cells including dendritic cells and monocytes (39, 40). However, to our knowledge, there is no evidence that NETs influence lymphocyte recruitment. Nevertheless, PAD4-mediated citrullination of lymphocyte chemoattractants CXCL10 and CXCL11 was demonstrated to impede the chemotaxis of T lymphocytes, suggesting a role of PAD4 for the regulation of chemokine activity (41). Thus, it is likely that increased lymphocyte content in atherosclerotic lesions may be explained by an increased chemotactic activity of non-citrullinated chemokines.

Notably, we found that PAD4 deficiency lowers lipid accumulation without affecting the number of intralesional macrophages after 10 weeks of HFD. In accordance to previously published work (12, 13), the majority of aortic macrophages were found to display an anti-inflammatory, M2-like phenotype characterized by low or absent Ly6C expression. However, PAD4 deficiency provoked increased content of M1-like macrophages (F4/80^+^Ly6C^high^), whereas the content of M2-like cells (F4/80^+^Ly6C^neg^) was significantly reduced. These unexpected findings strongly contradict previous reports demonstrating pro-inflammatory properties of PAD4 (23) and NETs (18). Previous work of our group revealed that NETs markedly diminish the expression of pro-inflammatory genes in murine M1-like macrophages and further increase the expression of M2-specific genes *in vitro* (26, 28). Consequently, here, mice with bone marrow cells-restricted PAD4 deficiency displayed higher content of M1-like cells and increased expression of vascular *CCL2* and *iNOS*. Indeed, PAD4 deficiency enhanced the expression of M1-associated markers *in vitro*, but did not affect macrophage polarization. Thus, the *in vivo* phenotype is likely due to the local environment including NETs deficiency. Besides, it may be assumed that recruited lymphocytes (42) and additional cell types secrete macrophage-polarizing factors, thus contributing to the imbalance of M1 and M2 macrophages. In this regard, CCL2, itself, which is mainly secreted by monocytes, macrophages, endothelial cells and SMCs (43), was reported to promote M1 polarization (44). However, the number of CCR2^+^ cells was not increased (unpublished observation).

We further confirm that *in vitro*-polarized M2 macrophages incorporate significantly more lipids than M1 macrophages (35), which is based on higher expression levels of the oxLDL receptor CD36 (45). Based on our findings, we propose that reduced lipid accumulation in mice with bone marrow cells-restricted PAD4 deficiency is due to the decreased number of M2-like macrophages (F4/80^+^Ly6C^neg^). In line with this assumption, additional reports have indicated that macrophage cholesterol accumulation associates with the suppression of M1 characteristic pro-inflammatory gene expression (46, 47). Cholesterol uptake was additionally found to trigger the unfolded protein response (48) resulting in lipotoxicity of M2-polarized macrophages (49). Consistent with these findings, diminished lipid accumulation in PAD4-deficient mice was associated with reduced necrotic core and lesion size. Moreover, as we previously reported that purified NETs enhance oxLDL incorporation by M1- and M2-like macrophages *in vitro* (28), NETs deficiency should be considered as an additional aspect resulting in reduced lipid uptake by PAD4-deficient cells in atherosclerotic lesions.

Lesion progression was associated with increased content of α-SMA-expressing cells and collagen production in *ApoE^–/–^* control mice. Importantly, PAD4 deficiency strongly diminished the content of α-SMA-expressing SMCs and consequently the content of extracellular matrix in atherosclerotic lesions at 10 weeks. Although one may expect that impeded collagen production might support plaque pathogenesis, it was already reported that SMCs-specific knockout of collagen type XV markedly attenuates lesion size, matrix content and fibrous cap formation (50). Therefore, impeded collagen production in PAD4-deficient lesions may attenuate lesion progression in atherosclerosis. As SMCs were evidenced to originate almost from the local vessel wall and all circulating progenitor cells were excluded as a source of plaque SMC (51), we raise the possibility that the recruitment of SMCs may be disturbed in PAD4 deficient mice. However, this question will require a future study on the basis of lineage-marked SMCs.

Though, this study has certain limitations. First, we did not investigate the impact of PAD4 on later, chronic disease stages and more research is required to elaborate whether PAD4 affects regression or stability of advanced lesions. Second, the low number of animals in some experiments may represent a further limitation of our study. Lastly, due to the lack of suitable tools for the specific suppression of NETs formation, their contribution to lesion development could not yet been elucidated.

Taken together, we demonstrate here for the first time, that bone marrow-restricted PAD4 deficiency impedes the development of atherosclerotic lesions, reduces lipid accumulation, necrotic core area and lesional collagen content. Surprisingly, these changes were accompanied by increased prevalence of intralesional M1-like macrophages, diminished content of M2-like cells and enhanced vascular inflammation.

On the whole, targeting of PAD4 does not prevent the development of atherosclerotic plaques but may represent a valuable therapeutic tool for amelioration of disease progression.

## Data availability statement

The original contributions presented in this study are included in the article/Supplementary material, further inquiries can be directed to the corresponding author.

## Ethics statement

This animal study was reviewed and approved by the Landesamt für Natur, Umwelt und Verbraucherschutz, Germany, Nos. 81-02.04.2018.A181 and 81-02.04.2018.A255.

## Author contributions

AP-G conceived and supervised the study, analyzed and interpreted data, and wrote the manuscript. AC performed animal experiments, flow cytometric analysis, and contributed to histological staining. NM performed histological staining and analyzed data. MZ supervised animal experiments, gave methodological support, and critically reviewed the manuscript. XX measured cytokine levels. TW supervised the study, interpreted data, and contributed to the writing of the manuscript. All authors contributed to the article and approved the submitted version.
