# Supplementary figures and images for: Peptidylarginine deiminase 4 deficiency in bone marrow cells prevents plaque progression without decreasing atherogenic inflammation in apolipoprotein E-knockout mice
Source: Front Cardiovasc Med. 2022 Nov 16;9:1046273. doi: 10.3389/fcvm.2022.1046273 (PMC9709396; doi:10.3389/fcvm.2022.1046273)

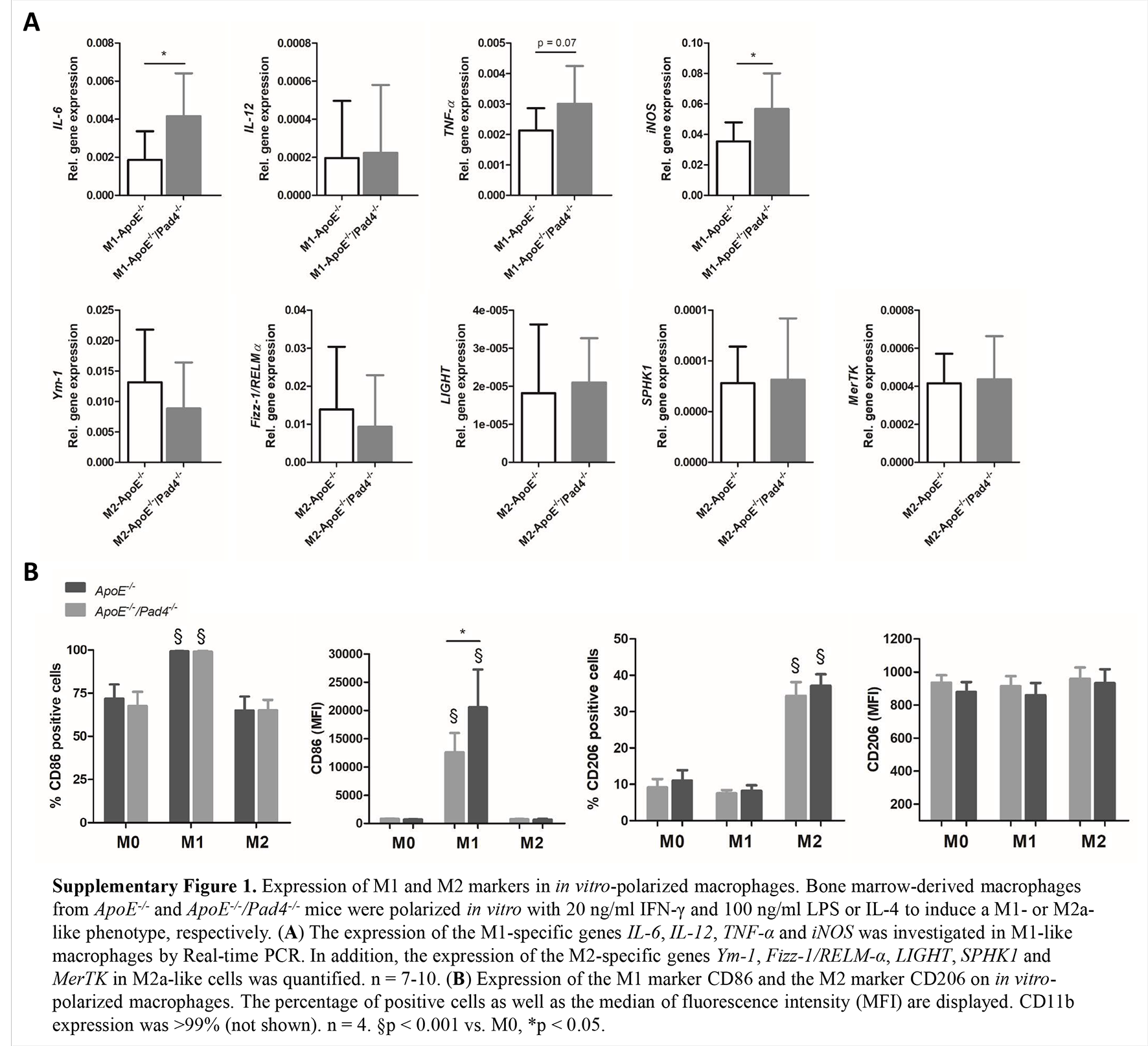

Supplement: Supplementary file 1 [file Image_1.TIF]
